# Identification of a Nine Immune-Related lncRNA Signature as a Novel Diagnostic Biomarker for Hepatocellular Carcinoma

**DOI:** 10.1155/2021/9798231

**Published:** 2021-01-05

**Authors:** Mengqin Yuan, Yanqing Wang, Qinqian Sun, Shiyi Liu, Shu Xian, Fangfang Dai, Li Zhang, Yaqi Fan, Feiyan Wang, Dongyong Yang, Yajing Zheng, Zhimin Deng, Wei Tan, Yeqiang Liu, Yanxiang Cheng

**Affiliations:** ^1^Department of Obstetrics and Gynecology, Renmin Hospital of Wuhan University, Wuhan, Hubei 430060, China; ^2^Department of Dermatopathology, Shanghai Skin Disease Hospital, Tongji University School of Medicine, Shanghai 200443, China

## Abstract

Hepatocellular carcinoma (HCC) ranks fifth among common cancers and is the second most common cause of cancer-related mortality worldwide. This study is aimed at identifying an immune-related long noncoding RNA (lncRNA) signature as a potential biomarker with prognostic value to improve early diagnosis and provide potential therapeutic targets for HCC patients. The subjects of this study were HCC samples with complete transcriptome data and clinical information downloaded from The Cancer Genome Atlas (TCGA) database. We then extracted the immune-related mRNA and lncRNA expression profiles. Based on the expression profiles of immune-related lncRNAs, we identified a nine-lncRNA signature that was related to the progression of HCC. The risk score was calculated based on the expression level of the nine lncRNAs of each sample, which divided patients into high-risk and low-risk groups. We found that the increased risk score was associated with a poor prognosis of HCC patients. To assess the accuracy of the survival model, we calculated a receiver operating characteristic (ROC) for validation. The curve showed that the area under the curve (AUC) for the risk score was 0.792. Besides, both principal component analysis (PCA) and gene set enrichment analysis (GSEA) were further used for functional annotation. We found that the distribution patterns were different between the low-risk and high-risk groups in PCA, and the underlying mechanism by which the nine lncRNAs promoted the progression of HCC involved an abnormal immune status. Finally, we analyzed the infiltration of twenty-nine kinds of immune cells and the activation of immune function in HCC using the ssGSEA algorithm. The results showed that aDCs, iDCs, macrophages, Tfh, Th1, Treg, and NK cells were correlated with the progress of HCC patients. And the immune functions including APC costimulation, CCR, check point, HLA, MHC class I, and Type II IFN responses were also significantly different between the high-risk and low-risk groups. In conclusion, our study identified a nine-lncRNA signature with potential prognostic value for patients with HCC, which could be used as a new biomarker for the diagnosis and immunotherapy of HCC.

## 1. Introduction

Hepatocellular carcinoma (HCC) is the most common primary liver cancer, accounting for approximately 90% of cases, and it has become a public health problem worldwide [1, 2]. Over the past few decades, the prevalence of chronic hepatitis, liver cirrhosis, the difficulty of early diagnosis of HCC, and the limited efficacy of available treatments are the major reasons contributing to the continuous increase of the incidence of HCC [3, 4]. It has become the second most common malignant tumor in the world, with an overall 5-year survival rate of less than 50% [5, 6]. The most commonly used biomarker for the early screening and diagnosis of HCC is alpha-fetoprotein (AFP), which has generated great controversy due to its limited sensitivity and specificity [7]. Therefore, it is imperative to identify more effective biomarkers for the early diagnosis and prognostic evaluation of HCC patients.

It is well known that the innate and adaptive immune systems play an essential role in mounting an anticancer response since they can recognize and destroy cancer cells [4]. Emerging evidence in cancer immunotherapy has confirmed that immunosuppression may significantly promote tumor growth and tumor niche formation [8]. Immunosuppression caused by adaptive changes of the tumor microenvironment during the stage of immune elimination may induce immune escape of tumor cells, and thus, accelerate tumor initiation and progression [9]. For instance, it was reported that smoking could promote immunosuppression by inhibiting immune cell infiltration, which may be related to the poor prognosis of HNSCC [10]. Besides, it was observed that the antigen-specific T cell response is significantly inhibited in ovarian cancer and is related to the occurrence of ovarian cancer [11]. Therefore, it is necessary to consider the immune-related factors related to the prognosis of patients with HCC.

Long noncoding RNAs (lncRNAs), noncoding RNAs with a sequence longer than 200 nucleotides that do not encode proteins, play a key role in the regulation of transcriptional and posttranscriptional events that impact cellular functions [12, 13]. Recent studies have shown that the dysregulation of lncRNAs contributes to tumor progression by regulating malignant biological behavior [14–16]. Wu et al. proved that the upregulation of lncRNA MER52A was associated with a poor prognosis of HCC and could serve as a biomarker and therapeutic target [17]. Lang et al. confirmed that lncRNA PCAT7 functions as a carcinogenic lncRNA that is related to bone metastasis status, and it acts as a potential therapeutic target for bone metastasis of prostate cancer [18]. Also, some limited evidence suggests that diverse lncRNAs are involved in the process of cancer immunity, such as immune activation, immune cell infiltration, and immune escape [19–23]. Jiang et al. demonstrated that lncRNA EGFR stimulated Treg differentiation in an EGFR-dependent manner and promoted the immune evasion of HCC [24]. At present, many studies have proposed that several lncRNAs could act as potential biomarkers for diagnosis, therapy, or prognostication of HCC [25–29]. However, the role of an immune-related lncRNA signature in the prognosis of HCC patients remains undefined. In this work, we developed an immune-related lncRNA signature with prognostic value to provide promising biomarkers and immune-related therapeutic targets.

## 2. Materials and Methods

### 2.1. Patient Data

The transcriptome profiles of 374 HCC samples and 50 cases of liver tissue adjacent to the tumor were obtained from The Cancer Genome Atlas (TCGA) database (https://portal.gdc.cancer.gov/). The clinical information including age, sex, vital status, TNM stage, and histological grade of the corresponding patients was obtained and assessed. Samples with overall survival (OS) less than 30 days were excluded from nontumorous death factors, including infection.

### 2.2. Identification of Expressed Immune-Related lncRNAs

First, the lncRNA and mRNA expression profiles were extracted separately. The molecular Signatures Database v7.0 (Immune system process M13664, Immune response M19817; http://software.broadinstitute.org/gsea/login.jsp) was implemented to screen the immune-related genes. Then, the immune-related lncRNA profiles were excerpted from the obtained immune-related genes based on the coexpression analysis. A group of immune-related lncRNAs was obtained by calculating the correlation coefficient between the expression levels of the lncRNA and immune-related genes (∣cor | >0.4, *p* < 0.001). Finally, univariate Cox regression was applied to identify immune-related lncRNAs related to the prognosis of HCC patients (*p* < 0.01) for further research. Hazard ratio (HR) was a variable related to OS. When HR > 1, immune-related lncRNA was defined as a risk factor for the prognosis of patients, and when HR < 1, it is a protective factor.

### 2.3. Construction of a Risk Signature

To assess the accuracy of the screened lncRNAs with the prognostic evaluation value, the risk score of each sample was calculated by the following formula [30, 31]:

(1) $$\begin{array}{c} \text{Risk score}=E\left(1\right)*C\left(1\right)+E\left(2\right)*C\left(2\right)+\cdots +E\left(n\right)*C\left(n\right). \end{array}$$

The risk score was calculated based on the expression level and the regression coefficient of the potential prognostic immune-related lncRNAs. *N* refers to the number of prognostic lncRNA. Ei suggests the relative expression level of the lncRNAs, and Ci denotes the lncRNA regression coefficient generated from a multivariate Cox regression analysis. Using the median score as the cutoff, 374 HCC patients in the TCGA database were divided into high-risk and low-risk groups for further study.

### 2.4. Bioinformatics Analysis

The survival R package was used to assess the relationship between the risk score and OS. The R package of “pheatmap” was performed to compare the expression levels of nine lncRNAs in different risk groups. The prognostic performance of the lncRNA signature was evaluated by the area under the curve (AUC) of the receiver operating characteristic (ROC) based on the risk score. We carried out principal component analysis (PCA) to analyze the profile expression characteristics of risk grouping samples and gene set enrichment analysis (GSEA; http://www.broadinstitute.org/gsea/index.jsp) to judge the biological course based on the different risk scores. And ssGSEA algorithm was carried out to analyze the infiltration of immune cells and the activation of immune function in HCC.

### 2.5. Statistical Analyses

In this study, the immune-related lncRNAs with significant prognostic value were screened by the cor.test and univariate Cox regression analyses. The multivariate Cox regression analysis was performed to establish a risk immune-related signature. The independent prognostic risk factors of HCC were identified using univariate and multivariate Cox regression analysis. Using R version 3.5.0 for all statistical analyses, statistical significance was defined as *p* < 0.05.

## 3. Results

### 3.1. Acquisition of Immune-Related lncRNAs

**Figure 1:**
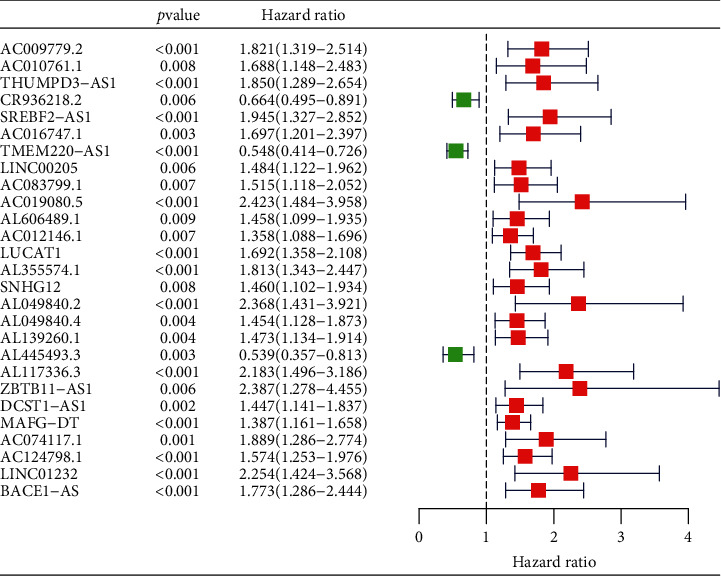
The twenty-seven immune-related lncRNAs with the prognostic value identified from univariate Cox regression analysis.

A total of 424 HCC samples were downloaded from the TCGA database, which included 374 HCC tissues and 50 liver tissue adjacent to the tumor. Their clinical characteristics are listed in Table 1. A total of 331 immune-related mRNAs were collected based on the Molecular Signatures Database v7.0, and then 338 immune-related lncRNAs were identified from the correlation analysis (∣cor | >0.4, *p* < 0.001). As shown in Figure 1, univariate Cox regression analysis (*p* < 0.01) was used to calculate the relationship between immune-related lncRNAs and OS, and the twenty-seven lncRNAs that were significantly associated with the prognosis of patients were screened out as candidate biomarkers for HCC patients. Among the twenty-seven lncRNAs, three were suggested to be protective factors (CR936218.2, TMEM220-AS1, AL445493.3, and HR < 1), and others were risk factors (HR > 1). Subsequently, a predictive signature of nine immune-related lncRNAs was established using the multivariate Cox regression analysis on the basis of the twenty-seven lncRNAs.  Table 2 illustrates the details of the nine lncRNAs (CR936218.2, SREBF2-AS1, TMEM220-AS1, LINC00205, LUCAT1, AL049840.2, AL139260.1, DCST1-AS1, and LINC01232) in the risk signature.

### 3.2. lncRNA Predictive Risk Score Analysis

**Figure 2:**
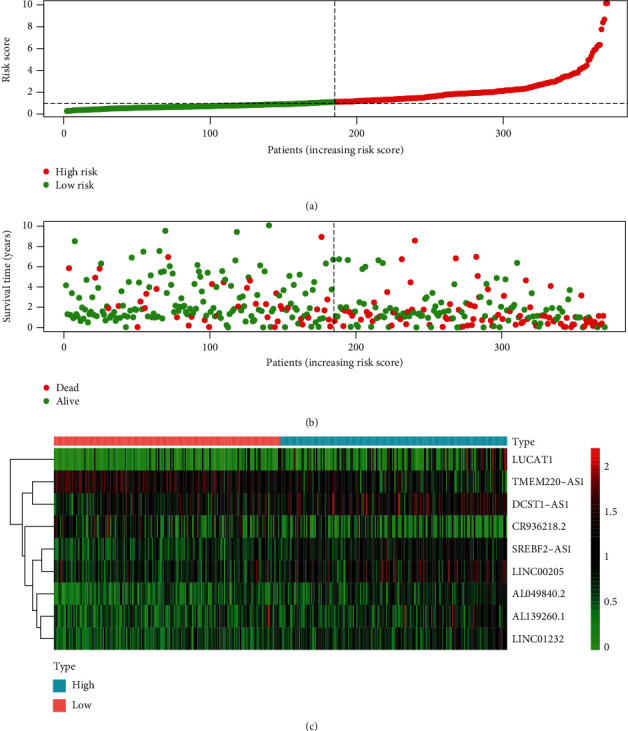
Immune-related lncRNA risk score analysis of HCC patients. (a) Risk score distribution of the 370 HCC patients; (b) HCC patients' survival status and time distributed by risk score; (c) heat map of the nine lncRNA expression profiles in HCC. Rows denote lncRNAs and columns represent patients. Green to red indicates a trend from low to high expression.

As the risk score was calculated based on the expression level of the obtained immune-related lncRNAs, we evaluated and ranked the risk scores of the patients. Then, by using the median risk score as the cut-off point, the patients were split into low-risk and high-risk groups (Figure 2(a)).  Figure 2(b) exhibits the survival status and time of the patients distributed by the risk score. We found that patients in the high-risk group had a worse survival status compared to those in the low-risk group. Besides, Figure 2(c) depicts the expression levels of the nine lncRNAs, and we can see that all lncRNA are differentially expressed. Compared with the low-risk group, the expression level of CR936218.2 and TMEM220-AS1 was lower in the high-risk group (*p* < 0.001, adjp < 0.001, and logFC < 0), while the expression level of SREBF2-AS1, LINC00205, LUCAT1, AL049840.2, AL139260.1, DCST1-AS1, and LINC01232 was the opposite (*p* < 0.001, adjp < 0.001, and logFC > 0).

### 3.3. Validation of the Nine Immune-Related lncRNA Signature

**Figure 3:**
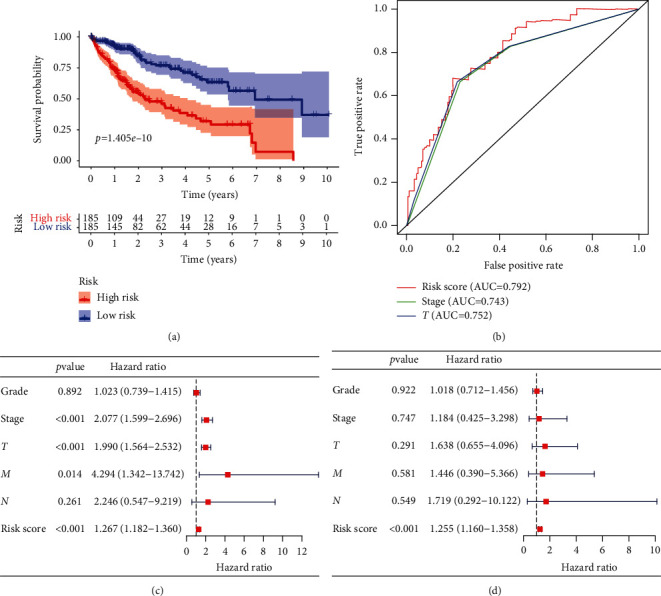
Validation of the nine immune-related lncRNA signature. (a) Kaplan-Meier analysis for the OS of HCC patients; (b) ROC curve of the signature in predicting the patients' OS; (c) univariate Cox regression analyses and (d) multivariate Cox regression analyses of the association between clinicopathological factors and OS of HCC patients.

To further confirm the potential correlation between the nine lncRNA signature and patient OS, Kaplan–Meier analysis was constructed. As shown in Figure 3(a), patients in the low-risk group had a significantly longer OS compared with those in the high-risk group (*p* = 1.405*e* − 10). A ROC curve was constructed to evaluate the accuracy of the lncRNA signature for predicting the patient's OS. The results show that the AUC value of the risk score model ROC curve was 0.792, indicating that the nine-lncRNA signature was effective in predicting the OS of the HCC patients (Figure 3(b)). We further used univariate and multivariate Cox regression analyses to determine whether the risk score was an independent prognostic indicator. The results suggested that four factors, including the risk score (HR = 2.077, *p* < 0.001), stage (HR = 1.990, *p* < 0.001), *T* status (HR = 4.294, *p* = 0.014), and *M* status (HR = 1.267, *p* < 0.001) were correlated with the OS (Figures 3(c) and 3(d)). These results indicated that the lncRNA signature could serve as a promising independent risk factor for predicting the outcomes of HCC patients.

### 3.4. Clinical-Pathological Characteristics of the Low-Risk and High-Risk Groups

**Figure 4:**
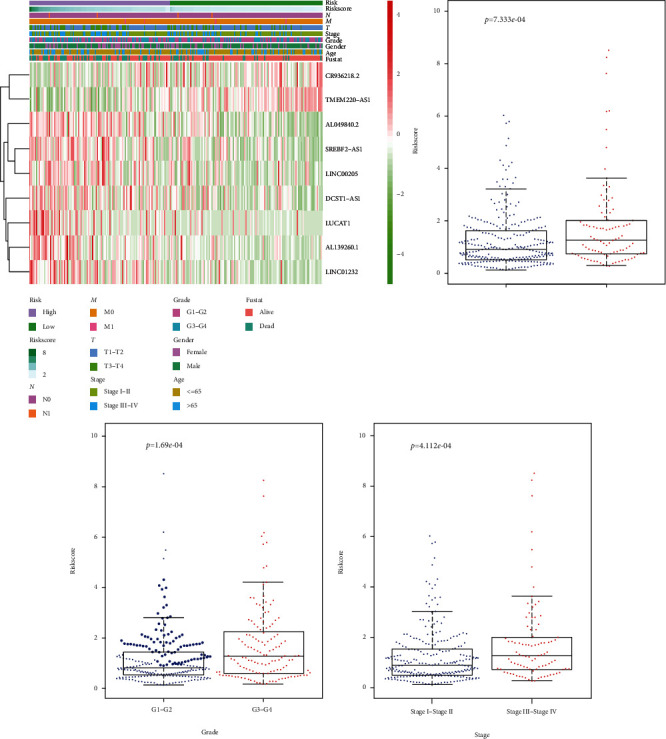
Associations between the signature risk scores and clinical-pathological characteristics. (a) Heat map of the clinical-pathological characteristics and lncRNA expression; (b, d) distribution of the risk scores in different cohorts stratified by the clinical-pathological characteristics.

The patterns distributed between the clinical-pathological characteristics and the risk score are depicted in a heat map, in which the clinical-pathological characteristics including grade, stage, *T* status, and fustat were significantly correlated with the high-risk group (Figure 4(a)). Moreover, by comparing the risk scores of samples with different clinicopathological conditions, we found that the clinical-pathological features, including grade, stage, and *T* status, were significantly correlated with the risk score (*p* < 0.001) (Figures 4(b) and 4(d)). Therefore, the nine-lncRNA signature was significantly associated with the progression of HCC.

### 3.5. Functional Assessment of the Low-Risk and High-Risk Groups

**Figure 5:**
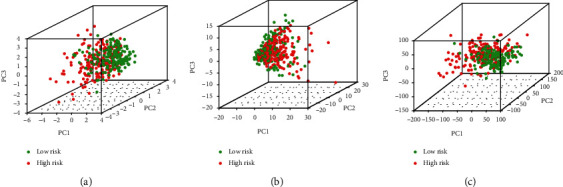
Distribution patterns of the low-risk and high-risk groups. PCA between low-risk and high-risk groups on the basis of (a) the nine immune-related lncRNAs signature, (b) the whole genome expression profile, and (c) the immune-related lncRNA expression.

**Figure 6:**
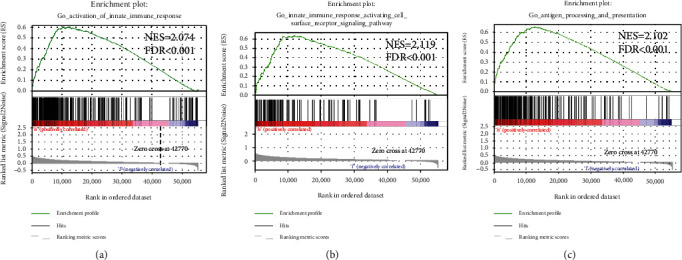
Differences in the immune-related phenotype between high-risk and low-risk groups.

**Figure 7:**
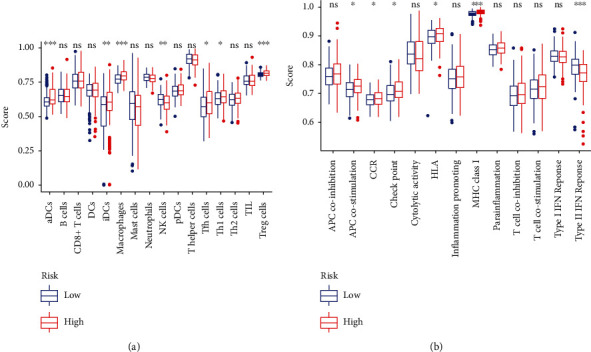
The differences in the infiltration of immune cells and activation of immune function in the low-risk and high-risk groups.

PCA was performed to distinguish the distribution patterns of the two groups. Based on the groups according to the risk score, the two groups were separated into two patterns, which indicated that the risk score could distinguish the distribution patterns of the two groups (Figure 5(a)). However, on the basis of the whole genome expression profile and the immune-related lncRNA expression, the PCA showed that the groups did not display a clear separation (Figures 5(b) and 5(c)). GSEA was further used to validate the functional annotation.  Figure 6 indicated that the immune responses, including activation of the innate immune response, innate immune response activating cell surface receptor signaling pathway, and antigen processing and presentation, were enriched in the high-risk group. Subsequently, the ssGSEA algorithm was performed to compare the infiltration of immune cells between high-risk and low-risk groups. Compared with low-risk groups, aDCs, iDCs, macrophages, Tfh, Th1, and Treg cells infiltration increased significantly in the high-risk group, while NK cells were the opposite (*p* < 0.05; Figure 7(a)). Additionally, immune-related functions including APC costimulation, CCR, check point, HLA, and MHC class I responses were enriched in high-risk groups, but Type II IFN response was enriched in low-risk groups (*p* < 0.05; Figure 7(b)). Altogether, the nine immune-related lncRNA signature was closely related to the immune status of HCC.

## 4. Discussion

HCC is a common digestive system cancer with the characteristics of high malignancy and high mortality. Despite the progress that has been made in researching HCC, its underlying molecular mechanisms remain to be elucidated. It is well known that effective biomarkers for early diagnosis and treatment are key factors that could improve the prognosis of patients with HCC [32].

Recent studies have reported that the cancer-associated immune response is a major factor in promoting the infiltration and metastatic dissemination of cells in the tumor microenvironment, which is closely correlated with high lethality and poor prognosis of cancer [4, 33]. Moreover, emerging evidences have illustrated that lncRNAs play a key regulatory role in cancer-associated immune responses, including the natural immune response and acquired immunity [34, 35]. For instance, Ma et al. suggested that significantly downregulated LncMX1-215 was related to the proliferation and metastasis of HNSCC by negatively regulating the expression of the immunosuppression-related molecules programmed death-1 ligand (PD-L1) and galectin-9 [36]. Wang et al. demonstrated that lncUCA1 overexpression contributed to the cells' immune escape and was closely associated with the prognosis of gastric cancer [19]. Consequently, immune-related lncRNAs could serve as biological markers for survival prognosis and be potential targets for cancer therapeutics.

Most of the previous studies have focused on single lncRNA expression patterns, but the complex molecular mechanisms of cancer introduce particular challenges [37, 38]. Fortunately, recent studies have indicated that multiple lncRNAs could be combined to improve the overall performance in predicting the outcomes of patients with cancer [39, 40]. Zhang et al. identified five novel plasma lncRNAs (TINCR, CCAT2, AOC4P, BANCR, and linc00857) that could be used as a diagnostic biomarker for gastric cancer detection [41]. Wu et al. proposed a five lncRNA signature (lncRNA-LET, PVT1, PANDAR, PTENP1, and linc00963) that could act as a biomarker for the diagnosis of clear cell renal cell carcinoma [42]. In this work, we aimed to construct an immune-related lncRNA signature to facilitate the prognostic evaluation of HCC patients.

To screen the immune-related lncRNAs that are associated with the prognosis of HCC, 374 HCC samples were enrolled, from which 338 immune-related lncRNAs were screened out for further research. In our study, a nine immune-related lncRNA signature (CR936218.2, SREBF2-AS1, TMEM220-AS1, linc00205, LUCAT1, AL049840.2, AL139260.1, DCST1-AS1, and linc01232) was considered to be a potential prognostic factor according to the univariate and multivariate Cox regression analyses.

The Kaplan-Meier curve showed that the lncRNA signature could distinguish between samples with a good prognosis and those with a poor prognosis (*p* < 0.001). We further found that the risk score was closely related to the malignant clinical-pathological characteristics of the samples. Compared with the low-risk group, the high-risk group showed a malignant clinical grade, stage, and *T* status. Our discovery indicated that the signature could be conducive to distinguishing the clinical prognostic characteristics including grade, stage, and *T* status of different patients, which was associated with the outcomes of the patients. Besides, the AUC value of the risk score model ROC curve was 0.795, which was larger than the AUC scores of the grade and stage curves, indicating that the signature was better and more accurate in discriminating patients with HCC compared to those with clinical-pathological characteristics.

Furthermore, GSEA analysis was performed to understand the related functional pathways of the nine-lncRNA signature. We confirmed that the immune status patterns were different between the two groups, and the immune responses were enriched in the high-risk group. Finally, we conducted a ssGSEA algorithm to identify the infiltration of immune cells and the activation of immune function in HCC by ssGSEA. The results showed that aDCs, iDCs, macrophages, Tfh, Th1, Treg, and NK cells are associated with the prognosis of HCC patients, and immune-related function including APC costimulation, CCR, check point, HLA, MHC class I, and Type II IFN responses are different significantly between the two groups. Therefore, our study may provide a nine immune-related lncRNA signature that serves as a novel biological biomarker for prognostication and treatment of HCC patients.

In our study, we constructed a nine immune-related lncRNA signature for early diagnosis and prognosis evaluation of HCC based on the TCGA database. However, it is worth noting that there are some limitations to our research. First, the predictive accuracy of the signature needs to be verified in additional studies to support or reject our findings.However, this effort is hindered by a shortage of HCC samples. The expression levels of these lncRNAs in HCC tissues need further experimental verification, such as via real-time PCR. Moreover, we need to conduct more in-depth functional studies on this nine-lncRNA signature in the future and verify the accuracy of the signature and its specific immunomodulatory mechanism in vivo and in vitro experiments.

## Figures and Tables

**Table 1:** Summary of clinical characteristics of HCC patients.

Characteristic	*n*
Age category	
<65/≥65/NA	224/152/1
Gender	
Male/female	255/122
Vital status	
Alive/dead	128/249
Tumor stage	
I/II/III/IV/NA	175/87/86/5/24
Tumor grade	
G1/G2/G3/G4/NA	55/180/124/13/5
*T* stage	
T1/T2/T3/T4/NA	185/95/81/13/3
*M* stage	
M0/M1/NA	272/4/101
*N* stage	
N0/N1/N2/N3/NA	257/4/116

**Table 2:** The immune-related lncRNAs predictors by multivariate Cox regression analysis.

lncRNA_symbol	HR	95% CI lower	95% CI upper	Coef	*p* value
CR936218.2	0.6446	0.4696	0.8850	-0.4391	0.0066
SREBF2-AS1	1.8487	1.1046	3.0941	0.6145	0.0194
TMEM220-AS1	0.6815	0.4969	0.9347	-0.3834	0.0174
LINC00205	0.7358	0.5004	1.0819	-0.3068	0.1188
LUCATI	1.3134	1.0370	1.6633	0.2726	0.0237
AL049840.2	2.4324	1.4315	4.1331	0.8889	0.0010
AL139260.1	1.4554	1.0194	2.0781	0.3753	0.0389
DCST1-AS1	1.5387	1.1867	1.9952	0.4310	0.0011
LINC01232	1.9340	1.1203	3.3388	0.6596	0.0179

## Data Availability

In this study, all data comes from publicly available databases and references to available data are included in the methodology section.
